# Viral Determinants of HIV-1 Macrophage Tropism

**DOI:** 10.3390/v3112255

**Published:** 2011-11-15

**Authors:** Christopher J. A. Duncan, Quentin J. Sattentau

**Affiliations:** Sir William Dunn School of Pathology, University of Oxford, South Parks Road, Oxford, OX1 3RE, UK; E-Mail: quentin.sattentau@path.ox.ac.uk

**Keywords:** macrophage, monocyte, transmitted/founder virus, tropism, HIV-1, evolution

## Abstract

Macrophages are important target cells for HIV-1 infection that play significant roles in the maintenance of viral reservoirs and other aspects of pathogenesis. Understanding the determinants of HIV-1 tropism for macrophages will inform HIV-1 control and eradication strategies. Tropism for macrophages is both qualitative (infection or not) and quantitative (replication capacity). For example many R5 HIV-1 isolates cannot infect macrophages, but for those that can the macrophage replication capacity can vary by up to 1000-fold. Some X4 viruses are also capable of replication in macrophages, indicating that cellular tropism is partially independent of co-receptor preference. Preliminary data obtained with a small number of transmitted/founder viruses indicate inefficient macrophage infection, whereas isolates from later in disease are more frequently tropic for macrophages. Thus tropism may evolve over time, and more macrophage tropic viruses may be implicated in the pathogenesis of advanced HIV-1 infection. Compartmentalization of macrophage-tropic brain-derived envelope glycoproteins (Envs), and non-macrophage tropic non-neural tissue-derived Envs points to adaptation of HIV-1 quasi-species in distinct tissue microenvironments. Mutations within and adjacent to the Env-CD4 binding site have been identified that determine macrophage tropism at the entry level, but post-entry molecular determinants of macrophage replication capacity involving HIV-1 accessory proteins need further definition.

## 1. Introduction

Macrophages are long-lived, terminally-differentiated tissue-resident cells capable of limited replication, that are phenotypically and functionally heterogeneous [1,2]. As innate immune sentinel phagocytes, their role involves clearance of pathogens, apoptotic cells and debris, and antigen presentation to T cells [1,2]. Macrophages are also important in HIV-1 pathogenesis. Infected macrophages in the brain are central to the development of HIV-1-associated neurological disorders (HAND) [3]. Macrophages remain productively infected by HIV-1, often without obvious cytopathic effects, forming long-lived virus reservoirs [4] with the potential to transmit HIV-1 to activated CD4+ T cells during cell-to-cell interactions [5,6]. Better understanding of macrophage tropism is therefore highly relevant to the design of strategies to reduce HIV-1 persistence and spread, and to eradicate viral reservoirs. Macrophage tropism is a feature conserved across lentivirus species [7]. The term tropism is principally used to define the target cell preference of the virus, although is frequently also used to describe chemokine co-receptor usage of HIV-1 [8].

The process of HIV-1 entry into macrophages has been well studied. C-type lectins [9], integrins [10] and heparan sulfate proteoglycans [11] may initially bind HIV-1, allowing subsequent engagement of CD4 and a chemokine coreceptor (usually CCR5 or CXCR4) by the envelope glycoprotein (Env) surface subunit gp120 [8]. CD4 binding induces a conformational change in gp120 that exposes the co-receptor binding site, and the co-receptor-gp120 interaction destabilizes the Env spike and triggers gp41-mediated membrane fusion [8]. Fusion may occur at the cell membrane or from within macropinocytic [12] or endosomal [13] vesicles.

Macrophage replication capacity of CCR5-using (R5) virus isolates can vary by up to 1000-fold *in vitro*, and many primary R5 isolates and most CXCR4-using (X4) isolates are unable to replicate in macrophages [14,15]. Thus the concept of tropism is both categorical (replication or not), and continuous (the spectrum of replication capacity). In the absence of a satisfactory definition of macrophage tropism in the literature, we define as macrophage tropic (mac-tropic), viruses capable of infecting macrophages *in vitro* in single-cycle or spreading infection assays using monocyte-derived macrophages (MDM). We consider viruses that do not replicate in single-cycle or spreading infection assays to be non-macrophage tropic. To conceptualise infection efficiency of mac-tropic isolates, we also introduce the term “macrophage replication capacity” (MRC). MRC is an arbitrary measure of replication depending on the assay used. Reference laboratory-adapted R5 HIV-1 isolates (including YU-2, BaL, and SF162) demonstrate higher MRC than most primary (‘field’) isolates or molecular clones obtained without amplification *in vitro*, and can provide a positive control for qualitative and relative quantitative assessment of tropism phenotypes of primary isolates. Together, the terms mac-tropic and MRC allow us to more clearly distinguish the complementary but distinct qualitative and quantitative aspects respectively of the tropism phenotype, which are often used interchangeably in the literature. Beyond such definitions, methodological considerations for obtaining primary viruses also warrant discussion in the context of tropism assessment.

Isolation of primary virus from cultured tissue samples or donor donor peripheral blood mononuclear cells (PBMCs) has the potential to drive adaptation to a specific cell-type that could confound assessment of the tropism phenotype, and may also select for viruses with more rapid *in vitro* replication kinetics that do not represent the dominant species *in vivo*. Bulk or limiting dilution PCR amplification of full-length molecular clones or *env* sequences avoids these issues, but is subject to *taq* polymerase errors (either mutations or recombination) as well as template resampling or bias in amplification due to primer selection [16]. These issues can be overcome by single genome amplification and sequencing (SGA) from a single template [17], although to date most studies have employed PBMC co-culture or bulk PCR amplification of *env* sequences.

The *in vivo* tropism of HIV-1 is understandably difficult to assess directly. Interpretation of surrogate markers of productive infection such as detection of proviral DNA in circulating monocytes [18] and tissue macrophages [19–21] can be confounded by host restriction factors, including those operating at the post-integration stage that regulate latency, as well as tissue-specific differences in macrophage HIV-1 permissiveness (see Section 5). Since intrinsic macrophage restriction factors for HIV-1 have been recently reviewed [22,23], we will focus here on the viral determinants of tropism at the entry and post-entry levels.

## 2. Beyond Co-Receptor Usage

HIV-1 tropism classification systems have gone through several iterations over the years (reviewed in [24]). When the principal co-receptors for HIV-1 were initially identified, it appeared that viral tropism for macrophages and R5 co-receptor preference, were closely correlated. Although this is generally the case, there are exceptions (reviewed in [24]); for example, it was noted that some CXCR4-using (X4) viruses could productively infect macrophages [25,26], whilst many R5 isolates could not [15], therefore indicating that the tropism phenotype is related to, but distinct from, co-receptor preference. A reasonable proposal to modify the co-receptor classification to incorporate macrophage tropism [24] has not been widely adopted; for clarity in this review we qualify cellular tropism alongside viral co-receptor preference.

## 3. Association with Disease Stage

Up to 50% of patients who progress to AIDS do so with a viral quasispecies that has switched co-receptor preference from R5 to X4 [24]. The remainder of individuals progress to AIDS without co-receptor switching, retaining the CCR5 preference of the initial transmitted virus(es). In these individuals the mac-tropism of R5 isolates amplified by PBMC co-culture has been associated with disease stage in cross-sectional studies [27,28]. R5 isolates from individuals with advanced HIV-1 infection or AIDS can display enhanced *in vitro* MRC compared to early infection isolates [27,28]. It is unclear whether this apparent association with disease progression may result from evolution of tropism with time, perhaps to counter progressive loss of target CD4+ T cells [29], or whether macrophage replication is a direct viral virulence mechanism in its own right [30]. Although only one study [31] has been specifically designed with the principal aim of assessing the dynamics of tropism with time, other longitudinal studies have provided further support for the proposed association between disease stage and mac-tropism/MRC for R5 isolates observed in cross-sectional studies [31–33]. Richards and colleagues [31] examined changes in R5 mac-tropism and MRC over time of clade B Envs amplified by PCR from proviral DNA or viral RNA in three subjects. Isolates were obtained periodically from the first 3 weeks to several years post-infection. The *env* sequences were cloned into an isogenic replication competent virus backbone. A highly divergent tropism phenotype was observed for early infection Envs, which may at least in part relate to the non-SGA method of sampling, which is prone to *taq* polymerase errors (see above). Clones from later stages of infection were more frequently mac-tropic, and in 2/3 subjects all late-stage clones were mac-tropic. In one subject who progressed to AIDS, MRC was highest at the time of AIDS diagnosis. Similarly Mascriota and colleagues [32] measured the *in vitro* tropism phenotype of primary isolates acquired longitudinally (ranging from 3–5 isolates) from 13 individuals by PBMC co-culture. Although the exact time of seroconversion was unknown, these patients were clinically classified as rapid, late or long-term non-progressors (LTNP) based on CD4+ T cell count and time to AIDS. The authors hypothesized that mac-tropism would decline over time in progressors. This hypothesis was discounted, but insufficient data were presented to identify whether there was, by contrast, a temporal increase in mac-tropism or MRC. A representative data plot (Figure 3 in [32]) with two subjects per group was presented. Consistent with the alternative hypothesis that mac-tropism increases with disease progression, most isolates from late or rapid progressors were mac-tropic. By contrast, 4/5 of the non-mac-tropic isolates were obtained from LTNP. There was also an increase in MRC over time in 1/2 rapid and late progressors, but no change in 2/2 LTNP. Finally, Etemad and colleagues [33] amplified the V1-V5 region of clade A *envs* by PCR from PBMC in early and chronic infection (from 8 subjects) and cloned these into an isogenic clade A *nef*-deleted replication competent backbone, and tested MRC. No consistent patterns were observed, which may possibly relate to the earlier disease stage of the chronic isolates (median 41 months post-seroconversion) tested here in comparison to other studies, or to the absence of *nef* (see Section 8). In 3/8 subjects, chronic isolates replicated more efficiently than early isolates, whilst in 4/8, no clear increase was seen. In 1/8 a decrease in MRC was observed. However the data from this individual may have been biased by the brief sampling interval between isolates (early isolate—6 months, chronic isolate—24 months) and the availability of only a single chronic isolate for comparative testing.

Overall, it appears that more isolates from individuals with advanced HIV-1 or AIDS are mac-tropic, and demonstrate higher MRC than earlier isolates [27,28,31], supporting the concept that tropism for macrophages evolves over time. However, the prospects for future prospective studies from early to advanced infection, which are required in order to better understand the dynamics of tropism, are limited by ethical constraints and moves towards earlier initiation of anti-retroviral therapy (ART) in chronic infection.

The selection pressures on viral populations *in vivo* that may influence this evolution in tropism are incompletely understood. CD4+ T cell attrition and associated B cell dysfunction, which occur as disease progresses [34], may influence the neutralizing antibody (NAb) response in advanced HIV-1. However, Richards and colleagues [31] demonstrated that the divergent mac-tropism identified in early PCR amplified Envs occurred prior to the development of NAbs. Intriguingly, when examining samples from the latest time point, NAbs from the individual with the fewest mac-tropic Envs had the greatest degree of homologous neutralization activity. One could speculate that this might suggest an inverse relationship between mac-tropism of viral isolates and the exposure to, and activity of, NAbs later in disease. However, this is far from established.

Tropism for macrophages has been proposed as a direct marker of pathogenesis in HIV-1 [30], although there are few *in vivo* data thus far in humans to support this hypothesis. In macaque models, increased monocyte turnover was observed in simian immunodeficiency virus (SIV) infection, and appeared to better predict disease progression than viral load or CD4 count [35,36]. Whether increased turnover of bone marrow and peripheral blood monocytes occurs in response to direct monocyte cell death in the blood or bone marrow, monocyte repopulation of dying tissue macrophages, or increased monocyte recruitment in response to local tissue inflammation following microbial translocation across immune-depleted gastrointestinal mucosa, is unclear. It is also unclear whether increased monocyte turnover also occurs in HIV-1 infection, although markers of monocyte and macrophage activation have been identified in HIV-1 patients in several studies (reviewed in [37]). In addition, these markers appear to persist despite peripheral blood CD4+ T cell recovery after ART [38]. An interesting report of HIV-1 controllers recently identified an as yet undefined partial restriction to HIV-1 replication in macrophages, which occurred at the post-entry level, suggesting that host cell range may also be involved, to some degree, in HIV-1 control [39].

## 4. Transmitted/Founder Viruses

Early reports proposed macrophages as a principal target for transmitted virus based on the mac-tropic phenotype of these isolates [40,41]. Mac-tropism was also proposed to be a component of the genetic bottleneck observed for mucosal transmission of R5 HIV-1 [42]. However, recent data challenge this paradigm. Studies in macaques indicate that mucosal T cells are the earliest targets of transmitted virus (reviewed in [43]), and transmitted viruses with reduced numbers of N-linked glycosylation sites were shown to have enhanced affinity for CD4+ T cells expressing the alpha-4 beta-7 integrin, a gut mucosal T cell homing marker [44]. Using the technique of single genome amplification (SGA), combined with a mathematical model of stochastic early virus evolution, transmitted sequences have been unequivocally proposed for diverse transmission routes of clade A-D viruses (reviewed in [16,45]). The SGA method involves amplification of a single template of RNA or proviral DNA, and provides more robust fidelity than traditional PCR amplification by avoiding *taq* polymerase errors, recombination, and template resampling (discussed above and reviewed in [16,45]). In 75–80% of mucosal transmissions, a single sequence (the transmitted/founder virus [T/F virus]) was proposed to be responsible for initiating infection [17,46,47], and this conclusion was recently supported by ultra-deep sequencing [48]. The remaining transmission events are initiated by a small number of T/F viruses, although multiple virus transmissions are significantly more frequent in the context of genital tract inflammation in the recipient [49], as well as by the rectal [50] or intravenous routes [51] of transmission. The preponderance of R5 T/F virus transmission also applies to horizontal infection from mother-to-child [52]. Overall these data demonstrate the genetic bottleneck at transmission, and implicate intact innate mucosal barriers to be of central importance in maintaining the bottleneck [42]. However, in conflict with the previous hypothesis that mac-tropism of T/F virus influences this genetic bottleneck, two studies report evidence of very low MRC of T/F viruses, testing three clade C [47] and one clade B [50] full-length infectious molecular clones (IMC) in MDM, despite equivalent replication kinetics in CD4+ T cells. Restriction at the level of entry may determine this phenotype, since a further two transmitted clade C Envs also tested displayed reduced MRC [53]. However the generality of entry dependence needs to be studied using a broader panel of Env-IMCs before firm conclusions can be drawn. In total, biological data on cellular tropism of only 6 full-length or *env* T/F sequences have been published to date. Unpublished data on additional clade B full-length molecular clones appear to be consistent with these findings, finding clade B T/F full length and Env-chimeric infectious clones are capable of limited replication, but at a low MRC compared to reference strains [54]. More recent unpublished data suggest that MRC may be higher for clade D T/F viruses [55], implying clade-specific differences in the tropism phenotype of T/F viruses, but clearly this is an evolving area. Although making definitive conclusions is premature, overall these preliminary data suggest that cell-free T/F viruses are unlikely to efficiently infect macrophages to high levels early in infection, indicating that evolution of MRC occurs downstream of transmission of the founder sequence.

In a further study, Isaacman-Beck and colleagues [53] found evidence of comparable mac-tropism but low MRC between donor and recipient clade C Envs isolated from transmitter pairs around the time of transmission. In agreement with studies of T/F viruses the authors concluded that transmitted Envs are not selected for macrophage replication, since the tropic phenotype of donor and recipient Envs was similar. However, the recipient *env* sequences were amplified (in all but two cases) without using SGA sequencing or a model of sequence evolution, and therefore most do not represent T/F viruses, but rather are comparable to the acute viruses studied by Richards and colleagues [31]. Moreover, spinoculation was used for *in vitro* infection assays, a method which enhances infection efficiency and has recently been implicated in enhanced CD4 and co-receptor expression due to cytoskeletal activation in T cells [56]. Therefore these data do not exclude the possibility of rapid evolution of mac-tropism from the founder sequence, but do highlight the need for acquisition of more phenotypic data on transmitted and early infection viruses. A major caveat of the published studies exploring the tropism phenotype of T/F viruses is that replication has been assessed only in MDM, rather than in more relevant tissue macrophages that have differential cellular permissiveness (see Section 5). In addition, alternative routes of macrophage infection by T/F viruses, such as cell-to-cell spread, which may play an important role *in vivo* [57], also remain unexplored.

## 5. Compartmentalization

Virus isolates from different tissue compartments demonstrate both intra- and inter-host mac-tropism diversity, with isolates from brain consistently demonstrating enhanced mac-tropism and replication capacity by comparison with isolates from other tissues such as blood and lymphoid tissues [15,19,58–68]. Genetic compartmentalization between tissues has been widely studied [69–78], and has also been noted between macrophages and T cells [18,79–81], although recent studies adopting the SGA approach suggest that clonal proliferation or amplification within tissues may have over-estimated previous measures of genetic diversity identified using bulk or limiting dilution PCR methods [82–86]. Earlier studies suggested T-cell associated virus was capable of replicating in macrophages *in vitro* [87]. In a study in which compartmentalized variants in blood monocytes were cloned and phenotyped, R5 mac-tropic viruses predominated, although R5X4 dual-tropic viruses were also isolated that infected both MDM and CD4+ T cells [88]. Within the CNS, a cross-sectional analysis associated compartmentalization with disease stage [89]. An interesting SGA-sequencing study of longitudinally-acquired CSF and blood samples provided evidence of independent and dynamic sequence variation in the CNS [90].

Compartmentalized viruses may evolve by stochastic variation within a spatially confined location, or as a result of adaptation to the available target cells, microenvironment and immune pressures [91]. Macrophages in different tissue sites are extremely heterogeneous [1], and display different levels of CD4 and coreceptor expression, responsiveness to activation and most importantly, permissiveness to HIV-1 infection [92]. For example, vaginal macrophages are more similar to MDM in phenotype and infectibility [93], whereas gut and alveolar macrophages are significantly less permissive [22,93]. It is unclear what mediates this heterologous permissiveness, although in the case of the well-characterized gut macrophages the restricted phenotype arises at both the entry and post-entry levels, and is associated with reduced expression of CD4 and CCR5, and reduced nuclear translocation of NF-κB [93,94]. The gut macrophage phenotype can be recapitulated *in vitro* by the application of intestinal stromal conditioned media to MDM and is dependent on TGF-β, highlighting the critical role of the tissue cytokine microenvironment in regulating macrophage permissiveness *in vivo* [93,94].

The majority of mac-tropic isolates with high MRC are brain-derived [48]. In the brain the most abundant target cells are perivascular macrophages and macrophage-lineage microglial cells, which unlike gut macrophages are highly infected despite expressing low levels of CD4 and co-receptors [64]. The alternative co-receptor CCR3 can also be used by some brain-derived Envs [95,96]. Moreover, host immune pressure from NAbs is contained by the blood-brain barrier [31]. Taken together, these data suggest that adaptation to tissue conditions, rather than stochastic variation, is the dominant influence in determining the viral tropism of compartmentalized variants [97]. However, the differential host immune pressures that may drive adaptation in different compartments remain unknown.

## 6. Influence of Recombination

Recombination occurs frequently during HIV-1 replication by switching of reverse transcriptase between heterologous RNA strands, which are present within individual cells as a result of multiple infection [98]. It has been suggested, using single fluorescently-tagged viruses, that recombination occurs more frequently in macrophages than T cells [99], although this finding has not been reproduced in other studies [100].

In a recent study employing SGA and sequencing of chronic isolates *in vivo*, inter-compartmental recombination events were shown to result in transfer of R5 Env determinants that dramatically altered R5 mac-tropism and MRC, conferring brain-like MRC to recombinant lymph node isolates [101]. As well as implicating Env as a key determinant of tropism, these data raise the possibility that in later infection, recombination events between R5 brain-derived viruses and other non-mac-tropic tissue-derived R5 viruses could expand intra-host macrophage infection capability. Estimates of the frequency of recombination were approximately 10–15% [101]. The mechanism(s) underpinning the necessary precursor events to intra-host recombination (such as inter-compartmental migration of cell-free or cell-associated virus, and subsequent multiple-virus infection of cells within the new compartment) need to be explored. As an efficient mode of viral dissemination [57], cell-to-cell spread more frequently leads to multiple single cell infections [102,103], which has recently been proposed as a mechanism permitting intermittent viral replication in T cells in the face of effective combination ART [102]. Cell-to-cell transmission may therefore also be implicated in increasing the likelihood of recombination events *in vivo*. Recent data provide evidence of transit of sequences between brain to periphery, occurring at the meninges [104]. Therefore, the brain may represent a reservoir of mac-tropic virus with high MRC, which can emerge in later stages of infection.

## 7. Molecular Determinants — Entry

Comparative studies of amino acid signatures in Env gp120 in high MRC *versus* non-mac-tropic isolates have identified several putative molecular determinants of R5 and X4 tropism for macrophages. These studies have predominantly utilized isolates obtained by co-culture with PBMC or amplified by PCR (with the inherent caveats described above), although the SGA-sequencing approach has been recently employed [101]. In most studies, the *env* sequence was cloned into pseudoviruses, and tropism defined as entry and replication in a single-cycle assay. Multiple single amino acid differences in the regions of R5 Env involved in CD4 binding principally distinguish between mac-tropic and non-mac-tropic isolates, as do incompletely characterized variations in the length and number of potential N-linked glycosylation sites (PNGS) in V1/2 regions of Env. In addition, there are some indications of an association between sensitivity to a limited number of CD4 binding site (CD4bs) NAbs and R5 mac-tropism/MRC [67,105].

### 7.1. CD4 Binding

Adaptation to efficiently enter cells expressing low surface CD4 has been observed in several studies using R5 mac-tropic brain isolates (reviewed in [64]), and R5 mac-tropism and/or MRC was shown to correlate with sensitivity to inhibitors of CD4-gp120 interactions [14,61,64,101,105,106], indicating that the macrophage tropic phenotype is strongly dependent on Env-CD4 binding affinity and/or exposure of the CD4 binding site. Molecular determinants include the N283 substitution in the C2 region, which enhances the binding affinity of gp120 to CD4 [107], and loss of the N-linked glycosylation site N386 in V4 [63]. N283 and N386D mutagenesis of non-mac-tropic R5 Envs conferred increased MRC [63]. N386 alone, or in combination with proximal residue R373 [108] was suggested to mediate resistance to the broadly neutralizing CD4bs antibody b12. These molecular determinants accounted for around 50% of the enhanced mac-tropism/MRC of the pseudotyped Envs tested. Additional determinants on the N-terminal flank of the CD4 binding loop within the CD4bs, that influence CD4bs exposure, have also been associated with enhanced macrophage tropism and altered sensitivity to the tetrameric soluble (s)CD4-based inhibitor PRO 542 [106]. A single E153G substitution within the gp120 V1 loop, distant from the CD4bs, was recently associated with enhanced R5 MRC, and was abrogated by the reciprocal mutation G153E [109]. The exact mechanism remains uncertain, but it probably involves increased exposure of the CD4bs due to a V1-induced conformational change in the V3 loop, which incidentally also conferred increased sensitivity to the CCR5 binding antibody 477-52D. Moreover, variations in the V1/V2 region were recently identified as conferring mac-tropism to recombinant viruses identified by SGA sequencing [101].

Although mac-tropic Envs display increased sensitivity to inhibitors of CD4 binding, implicating the CD4bs [14,61,64,101,105,106], the relationship between the R5 Env properties of mac-tropism or MRC, and neutralization sensitivity to NAbs targeting the CD4bs, is less clear. Originally, Gray and colleagues demonstrated an association between R5 mac-tropism/MRC and sensitivity to b12 [14], confirmed separately by Dunfee and colleagues [67]. However, by contrast with previous studies, Dunfee and colleagues did not identify an association between R5 MRC and sensitivity to sCD4 [67]. N386D mutagenesis of the R5 reference strains YU2 and JRFL enhanced MRC by 1–2 orders of magnitude, and increased sensitivity to b12 by approximately 2-fold [67]. However, not all studies confirm the association with b12 sensitivity. For reasons as yet unclear, Brown and colleagues identified an inverse association with either b12 or 2G12 sensitivity [101] despite demonstrating a clear association between sensitivity to sCD4 and MRC. Variation in mac-tropism also emerged prior to autologous NAb responses in the study by Richards and colleagues [31], suggesting that the properties of NAb sensitivity and mac-tropism may be distinct, although there appeared to be a temporal association between reduced MRC and higher NAb activity in later isolates. Taken as a whole, the data are inconclusive with regard to an association between NAb sensitivity and mac-tropism or MRC, and more studies are required.

### 7.2. Co-Receptor Binding

Similar to the differential interaction with CD4, variable sensitivity to CCR5 inhibitors has also been observed for mac-tropic R5 Envs (reviewed in [64]). Reduced sensitivity to fusion inhibitors of chronic, compared to early R5 Env isolates spanning V1-5, was not associated with enhanced MRC despite increasing the efficiency of productive infection in cell lines and PBMCs, for reasons which are unclear [33]. However, a recent study employing a luciferase reporter system and a panel of R5 *env* sequences identified a correlation between entry efficiency in CCR5^low^ cell lines with stable CD4 expression, and MRC, suggesting the mac-tropic Envs could more efficiently use low levels of co-receptor [110]. Inhibition by the CCR5 entry inhibitor maraviroc was also reduced in the mac-tropic R5 Envs, implying that conformational changes that enhanced R5 binding also impaired small molecule inhibition. Duenas-Decamp and colleagues [106] observed V3 loop determinants of R5 mac-tropism. They suggested that a conformational change in the V3 loop increased exposure of the CD4bs.

Whether enhanced co-receptor binding is linked to the macrophage tropism phenotype for X4 Envs has been, until very recently, unexplored. Although the majority of mac-tropic brain-derived Envs are R5, some brain isolates can differentially use either CCR5 or CXCR4 for entry into macrophages. Non-brain R5X4 viruses, on the other hand, use X4 preferentially for macrophage entry [111]. In an elegant mutagenesis study of an unusual brain-derived R5X4 virus it was shown that S306 in V3 conferred enhanced R5-entry via decreased dependence on the N terminus of CCR5, whereas in contrast R306 conferred enhanced X4-entry via decreased dependence on the N-terminus of CXCR4. This study provides a putative molecular determinant of co-receptor preference for a mac-tropic R5X4 envelope [111].

Recently, promising additional determinants were identified that conferred mac-tropism to more unusual X4-using mac-tropic Envs [112]. These included amino-acid variants at position 326 in V3, and at positions 261 and 263 within the gp41 interactive region of gp120. The presence of Ile 326 was critical for mac-tropism across divergent X4 Envs. These X4 Envs were sensitive to large deletions in the N-terminus of the chemokine coreceptor CXCR4, suggesting that co-receptor binding and mac-tropism for X4-using viruses are linked.

## 8. Molecular Determinants — Post Entry

Humans have co-evolved with retroviruses, and some 8% of the human genome (more numerous than the protein coding regions) is thought to comprise redundant retroviral elements [113]. There are a number of host restriction factors that partially regulate cellular permissiveness to HIV-1 infection post-entry, some of which are type I IFN-inducible (e.g., Tetherin/BST-2 and the APOBECs). Those relevant to macrophage tropism are reviewed in [22]. The fact that restriction factors are unable to prevent ongoing HIV-1 replication in the host indicates that the virus has evolved successful strategies to overcome such restrictions, which primarily involve the non-structural accessory proteins of HIV-1, such as Vif (targets APOBEC3G) and Vpu (targets tetherin) [114]. Viral infectivity is also associated with other regulatory proteins such as Nef and Tat. HIV-1 accessory proteins are the focus of a great deal of research, most of which falls out of the scope of this article (reviewed in [114] and [115]). Here we discuss specifically the role of the HIV-1 accessory proteins such as Tat, Nef, Vif and Vpu in determining MRC.

### 8.1. Tat

The viral trans-activator protein Tat regulates viral gene expression, through interactions with the HIV-1 transactivation response element (TAR) RNA [116]. Reduced *tat* mRNA expression in MDM has been shown to correlate with reduction in steady-state R5 virus production [117], which can be recovered by the addition of exogenous Tat protein [117]. Alterations of *tat* expression have been linked to changes in the balance of host cellular RNA processing factors, rather than a reduction in *tat* mRNA stability [118]. Truncation mutants of *tat* (wherein the second coding exon is deleted) have highlighted the importance of the second coding exon for MRC in MDM [119] despite preserved single-cycle replication of these *tat* mutants in reporter cell lines, which implies the second coding exon may have additional activity in MDM separate from transcriptional activation [119]. In a study of LTR sequences, amplified by nested PCR and cloned into luciferase-expressing pseudoviruses from patients during progression to AIDS, there was an increase in the *in vitro* single-cycle transcription activity of late stage HIV-1 LTR in various target cells (including MDM) in a subset of patients, suggesting the enhanced replication capacity of isolates observed later in disease (see Section 3) may be partially mediated by LTR transactivation activity [120]. However, compartmentalization of PCR-amplified *tat* sequences in neural *versus* lymphoid tissues was not convincingly demonstrated in a recent study, and no enhancement of LTR transactivation activity by brain-derived *tat* sequences in patients with HIV-associated dementia (HAD) was observed [121], again suggesting that if *tat* is important in determining MRC this function may be distinct from LTR transactivation. In keeping with this hypothesis, exogenous Tat protein has been shown to enhance MRC through upregulation of the IL-7R, leading to enhanced IL-7R signaling in response to IL-7 [122]. Whether this or a similar mechanism, rather than LTR transactivation, accounts for the enhancement of MRC by *tat* remains to be conclusively determined.

### 8.2. Vif

The viral infectivity factor (Vif), as the name suggests, enhances infection. *vif*-deleted viruses are either unable to establish spreading infections in MDM [123,124], or can only sustain very low levels of MRC [125]. The activity of Vif appears to be dominated by its targeting of the host restriction factor apoplipoprotein B mRNA-editing, enzyme-catalytic, polypeptide-like 3G (APOBEC3G) [126]. APOBEC3G is packaged into progeny virions and restricts infectivity by inducing G-to-A hypermutations in viral DNA in subsequent rounds of infection (reviewed in [127]). Additional non-editing roles for APOBEC3G in regulating viral infectivity have been described [127]. Paradoxically, in long-term MDM culture, reduced virion infectivity over time was correlated with a reduction in APOBEC3G expression (independent of cytotoxicity), suggesting that Vif may also target an additional host factor(s) that may impact on virion infectivity, possibly through impaired processing/assembly of Gag capsid [128]. However, few studies have examined specific molecular determinants of Vif in relation to macrophage replication.

### 8.3. Nef

The negative factor (Nef) is a 25-27kDa myristoylated protein with multiple functions in mediating immune evasion and pathogenicity [114,115] that impact upon *in vivo* disease progression [129–131]. Nef-deleted viruses display impaired MRC *in vitro* [132,133], and *in vivo* have been linked to long-term non-progression (LTNP), for example in recipients of blood-products contaminated with *nef*-deleted HIV-1 [134]. However, evidence is lacking to relate the pathogenicity of the *nef*-deleted virus with alterations in macrophage tropism phenotype: a longitudinal analysis of R5 blood isolates (PBMC-isolated) in one long-term survivor who eventually progressed to AIDS did not demonstrate an increase in the low level of MRC during disease progression [135].

To examine the role of *nef* in MRC, a mutagenesis study was conducted using the R5 macrophage-tropic reference strain SF162 [136]. Deletion of *nef* resulted in delayed replication kinetics and reduced MRC, in keeping with previous data. Not surprisingly, significant inter-donor variation in MRC was observed, but there was a donor-dependent association between reduced MRC and amino acid mutations in the PXXP motif [136]. Interestingly mutations in *nef* associated with loss of CD4 down-regulatory function were associated with reduced MRC [136], in contrast to an earlier study in cell-lines [137] but in keeping with studies in primary T cells [138] and *ex vivo* lymphoid tissue [139]. CD4 down-regulation of infected cells has been proposed as a mechanism involved both in the efficient release of mature budding virions from the host cell membrane [140,141] and in enhancing incorporation of gp160 into the viral envelope [142]. Recent data indicate that HIV-1 virions in infected macrophages may bud from a plasma membrane-bound, surface-connected “quasi-intracellular” compartment [143–145], suggesting that these mechanisms of CD4 down-regulation could be involved in efficient Nef-mediated virus particle release from macrophages. But whilst compartmentalization of *nef* sequences amplified by PCR from neural and blood/lymphoid tissue was observed in a recent study in patients with HIV-associated dementia [146], the CD4 down-regulation activity of these *nef* sequences was conserved in both CNS and blood/lymphoid sequences, despite clearly distinct mac-tropism [146]. Molecular sequence determinants of MRC in *nef* were not assessed. This study and others described above suggest that determinants of CD4 down-regulation may not be causally associated *in vivo* with the macrophage tropism phenotype. Multiple additional functions of *nef* unrelated to MRC may impact pathogenesis [114]. However, in a recent systematic analysis of *nef*-mutations in long-term non-progressors, no consistent molecular signatures were associated with LTNP, and variation in *nef* was suggestive of rapid sequence evolution rather than deterministic mutations favoring attenuated pathogenicity [147]. Further work is required to establish whether the well-established molecular determinants of *nef* functional activity are linked to the macrophage tropism phenotype, and to identify specific molecular determinants in *nef* mediating MRC.

### 8.4. Vpu

HIV-1 viral protein U (Vpu) is a 16kDa protein produced late in the viral replication cycle. The two main functions of Vpu are to counteract the host type-1 IFN-induced restriction factor tetherin/BST-2 to allow viral particle release [148,149] and to degrade intracellular CD4 [114]. Viruses deleted for *vpu* demonstrate low MRC in macrophages [150], which has been associated with increased tetherin/BST-2 expression and reduced particle release [151]. Counteraction of tetherin is more important for *in vitro* replication in MDM than in lymphocytes or in *ex vivo* lymphoid tissues [151]. Interestingly, some R5 isolates with high MRC (e.g., AD8 and YU-2) have start codon mutations in *vpu*, which were previously believed to be redundant and could perhaps be compensated by Env [152], however a study of chimeric *vpu*-deleted viruses expressing YU-2 and AD8 Envs, which had low MRC *in vitro*, suggested Env cannot overcome the loss of Vpu [153]. In addition, a role for *vpu* in reducing virus particle release independent of tetherin downregulation in some cell-lines (but not macrophages) has been identified [154]. There is no current satisfactory explanation for the curious paradox of high MRC isolates with start codon mutations in *vpu*, but it is tempting to speculate on a more complex role for tetherin in efficient macrophage replication.

### 8.5. Vpr/Vpx

The lentiviral accessory viral proteins R and X (Vpr and Vpx, which have a common genetic origin) are also involved in determining tropism for macrophages. The role of Vpr/Vpx has been recently reviewed [155], but recent progress warrants acknowledgement here. Vpr is unique to HIV-1, and functions by triggering G2 cell cycle arrest and regulating the nuclear transport of the pre-integration complex. Deletion of Vpr reduces MRC *in vitro*, although additional roles are also proposed for Vpr, and its *in vivo* relevance is unclear [155]. Vpx is present in HIV-2 and some SIV strains, but not HIV-1, and is indispensable for replication in myeloid cells such as macrophages and dendritic cells [155]. When packaged with HIV-1, Vpx also enhances HIV-1 mac-tropism [156,157]. Recent studies have identified a host SAM-domain HD-domain containing protein 1 (SAMHD1) as a target for Vpx-mediated proteosomal degradation, which overcomes a post-entry block to reverse transcription in myeloid cells [158,159]. SAMHD1 [158], like the exonuclease TREX1 [160] may be responsible for innate sensing of viral DNA, and viral targeting of SAMHD1 may possibly contribute to viral innate immune evasion, although this has not been specifically addressed. Since multiple post-entry restriction factors operate in macrophages [22] it is highly probable that many other molecular determinants of mac-tropism have yet to be identified.

## 9. Conclusion

**Figure 1.**
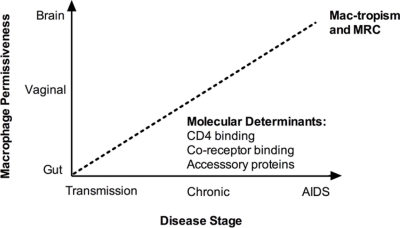
Determinants of macrophage tropism (mac-tropism) and macrophage replication capacity (MRC). Theoretical schematic representation of the interacting spectrum of HIV permissiveness between different tissues (from low to high on y-axis), and tropism of isolates from various stages of infection (from low to high on the x-axis), in determining mac-tropism and macrophage replication capacity (MRC) of isolates from various tissues. A linear relationship is entirely speculative, as indicated by the dotted line. The key molecular determinants of mac-tropism concern receptor-binding efficiency (both CD4 and chemokine co-receptor), whilst post-entry the accessory proteins (e.g., Vif, Nef, Vpu, *etc.*) mediate MRC. AIDS = acquired immunodeficiency syndrome.

Here we have reviewed recent studies concerning viral factors that modulate the macrophage tropism phenotype of HIV-1 (see Figure 1). We continue to have an incomplete understanding of the viral determinants of tropism for this important target cell, such as which host and virus factors drive the apparent compartmentalization of isolates between brain and other tissues, and what molecular mechanisms confer efficient HIV-1 replication post-entry. It should also be acknowledged that to date, most data on tropism phenotype comes from *in vitro* assays using MDM, which may not be representative of certain tissue macrophages in terms of permissiveness to viral infection. However, significant progress has been made, particularly regarding putative amino-acid changes determining the CD4 binding efficiency of R5 En*vs.* Moreover, improved sequencing techniques will allow a systematic characterization of the biological phenotype of clinically-relevant transmitted and early infection viruses, and should allow questions to be addressed regarding the mediators of tropism evolution and its role in disease pathogenesis. More work is required to delineate specific molecular determinants of macrophage replication subsequent to viral entry.
